# Tumor-derived extracellular vesicles disrupt the blood–brain barrier endothelium following high-frequency irreversible electroporation

**DOI:** 10.1038/s41598-024-79019-5

**Published:** 2024-11-18

**Authors:** Kelsey R. Murphy, Kenneth N. Aycock, Spencer Marsh, Alayna N. Hay, Ilektra Athanasiadi, Shay Bracha, Christine Chang, Robert Gourdie, Rafael V. Davalos, John H. Rossmeisl, Nikolaos G. Dervisis

**Affiliations:** 1 Department of Biomedical and Veterinary Sciences, Virginia-Maryland College of Veterinary Medicine, Blacksburg, VA USA; 2 Department of Biomedical Engineering and Mechanics, Virginia Tech, Blacksburg, VA USA; 3 Fralin Biomedical Research Institute at Virginia Tech Carilion School of Medicine, Virginia Tech, Roanoke, VA USA; 4 Center for Heart and Reparative Medicine Research, Virginia Tech, Roanoke, VA USA; 5 Department of Small Animal Clinical Sciences, Virginia-Maryland College of Veterinary Medicine, Blacksburg, VA USA; 6 Department of Veterinary Clinical Sciences, College of Veterinary Medicine, The Ohio State University, Columbus, OH USA; 7 Translational Biology Medicine and Health Graduate Program, Virginia Tech, Roanoke, VA USA; 8 Department of Emergency Medicine, Virginia Tech Carilion School of Medicine, Virginia Tech, Roanoke, VA USA; 9 ICTAS Center for Engineered Health, Virginia Tech, Kelly Hall, Blacksburg, VA USA; 10 Department of Internal Medicine, Virginia Tech Carilion School of Medicine, Roanoke, VA USA; 11 Department of Veterinary Small Animal Clinical Sciences, College of Veterinary Medicine, Texas A&M University, College Station, TX USA; 12 Department of Veterinary Clinical Sciences, College of Veterinary Medicine, Institute for Cancer Research, Purdue University, West Lafayette, IN, USA; 13 Department of Clinical Sciences, Cummings School of Veterinary Medicine, Tufts University, North Grafton, MA, USA

**Keywords:** CNS cancer, Cancer microenvironment, Cancer therapy, Cancer therapy, Blood-brain barrier

## Abstract

High-frequency irreversible electroporation (H-FIRE), a nonthermal brain tumor ablation therapeutic, generates a central tumor ablation zone while transiently disrupting the peritumoral blood–brain barrier (BBB). We hypothesized that bystander effects of H-FIRE tumor cell ablation, mediated by small tumor-derived extracellular vesicles (sTDEV), disrupt the BBB endothelium. Monolayers of bEnd.3 cerebral endothelial cells were exposed to supernatants of H-FIRE or radiation (RT)-treated LL/2 and F98 cancer cells. Endothelial cell response was evaluated microscopically and via flow cytometry for apoptosis. sTDEV were isolated following H-FIRE and RT, characterized via nanoparticle tracking analysis (NTA) and transmission electron microscopy, and applied to a Transwell BBB endothelium model to quantify permeability changes. Supernatants of H-FIRE-treated tumor cells, but not supernatants of sham- or RT-treated cells, disrupted endothelial cell monolayer integrity while maintaining viability. sTDEV released by glioma cells treated with 3000 V/cm H-FIRE increased permeability of the BBB endothelium model compared to sTDEV released after lower H-FIRE doses and RT. NTA revealed significantly decreased sTDEV release after the 3000 V/cm H-FIRE dose. Our results demonstrate that sTDEV increase permeability of the BBB endothelium after H-FIRE ablation in vitro. sTDEV-mediated mechanisms of BBB disruption may be exploited for drug delivery to infiltrative margins following H-FIRE ablation.

## Introduction

The annual mortality rate of malignant brain tumors is approximately 4.5 per 100,000 people^1^, which encompasses both primary brain tumor- and brain metastasis (BM)-related mortality. Diffuse gliomas are the most common malignant primary brain tumor in adults, accounting for approximately 80% of all malignant cases^2^. Grade IV glioma, or glioblastoma (GBM), remains invariably lethal^2^ despite decades of research and a trimodal therapeutic approach consisting of maximum safe surgical resection, temozolomide chemotherapy, and radiation therapy^3^. Despite this treatment regimen, GBM remains universally fatal due to invasive malignant cells which preclude adequate surgical resection margins, leading to tumor recurrence^4^. Further, the peritumoral blood–brain barrier (pBBB) shields these infiltrative cell populations from effective delivery of chemotherapeutics, further contributing to tumor recurrence^5^.

Equally urgent, brain metastases (BM) represent the most common brain neoplasms in adults, occurring at ten times the frequency of primary brain tumors^6^ and comprising more than half of all brain tumors^5^. An estimated 20–40% of patients with cancer develop BM during the course of their illness^7^, most commonly arising from lung, breast, skin, and kidney cancers^8–10^. Treatment options include a combination of surgical resection, whole-brain radiotherapy (WBRT), stereotactic radiosurgery (SRS), or chemotherapy^11^. Treatment is largely palliative due to surgical limitations for resection of multiple BM, questionable efficacy of WBRT^12^, and protection of BM from chemotherapeutics by the blood–brain barrier (BBB)^5^. BM are often considered end-stage disease with a median survival of up to six months with treatment^5^.

In both primary brain tumors and BM, tumor cell resistance to chemotherapy and radiotherapy (RT)^13^ and RT-associated side effects^12,14^ further hinder effective treatment. In the case of GBM, there have been only four FDA approvals for systemically administered therapies in the past three decades^15^. The limited therapeutic progress in recent decades and minimal survival benefits of current BM and GBM treatment options necessitate the development of minimally-invasive and selective therapeutic techniques that address the treatment challenges of BBB insulation of malignant cells from systemic therapeutics, malignant invasion into healthy brain parenchyma, and therapeutic resistance.

Energy-based tumor ablation therapeutics demonstrate promise for the improvement of the current brain tumor treatment regimen. High-frequency irreversible electroporation (H-FIRE) is a novel, minimally-invasive, nonthermal tumor ablation modality. During H-FIRE tumor ablation, needle electrodes are inserted into the neoplastic tissue and deliver high-frequency bipolar electric pulses directly into target tumor tissue. This elevates transmembrane potential of tumor cells, resulting in the irreversible formation of cell membrane pores and the induction of cell death within a sharply demarcated treatment volume^16^. H-FIRE is able to produce uniform and predictable ablations with negligible thermal effects while significantly reducing muscle contractions observed during standard irreversible electroporation^17^. For the treatment of brain neoplasms, H-FIRE treatment is delivered minimally-invasively through craniotomy defects, and treatment times are on the order of minutes per cubic centimeter of tissue^18^. H-FIRE is delivered under ultrasound-, computed tomography (CT), and magnetic resonance (MR)-guidance, allowing for intraoperative assessment of electrode placement and post-ablation monitoring. Further, pro-inflammatory lymphocytes have been identified in the reactive zones surrounding treated tumor volumes, suggesting the induction of an inflammatory immune response after tumor ablation^19^. H-FIRE appears to provide clinical efficacy in the treatment of primary brain tumors in dogs while sparing surrounding healthy brain tissue, highlighting the potential of H-FIRE as a minimally invasive ablative option for people^18^.

In addition to brain tumor ablation, H-FIRE focally and transiently disrupts the pBBB in a penumbra surrounding the ablated tumor volume, which can be exploited to deliver systemically administered therapeutics to the infiltrative tumor cells beyond the borders of ablation^18,20,21^. Interestingly, both H-FIRE and RT, a standard treatment modality for primary brain tumors and BM, induce such pBBB disruption following tumor treatment^18,21–25^. In the case of RT, the pBBB disruption following treatment is attributed to direct exposure of pBBB cells to radiation and the subsequent induction of apoptosis, ultrastructural changes, and senescence in BBB endothelial cells^22,23^. However, the mechanisms responsible for this pBBB disruption after H-FIRE are not well understood. Similarly to RT-induced pBBB disruption, substantial evidence demonstrates the direct effects of pulsed electric fields on BBB permeability in healthy brain tissue^21,26–30^, which may contribute to the overall mechanism of pBBB disruption following H-FIRE brain tumor ablation. However, the role of the post-ablation tumor secretome in this mechanism of pBBB disruption is understudied. A comprehensive understanding of the interactions between the H-FIRE-induced secretome and its interaction with the pBBB is paramount for the rational design of multimodal treatment approaches utilizing H-FIRE and improved peritumoral delivery of systemic therapeutics.

Recent studies suggest a role of bystander effects involving the release of extracellular signaling factors, such as small tumor-derived extracellular vesicles (sTDEV), from ablated tumor cells^31,32^. Many of these signaling mediators have been otherwise shown to alter BBB permeability and physiology in healthy brain environments^33–36^. However, the role of these bystander effects in pBBB disruption when brain tumors are ablated with H-FIRE is incompletely understood. Sheybani et al. demonstrated that treatment of glioma cells with focused ultrasound hyperthermia ablation augments the release of small tumor-derived extracellular vesicles (sTDEV)^37^. sTDEV carry complex bioactive cargo, which allows them to facilitate diverse intercellular communication between tumor cells and their local and systemic microenvironments via transfer of the vesicular cargo from parent tumor cells to local and distant stromal cells through endocytosis or fusion of the vesicle with the plasma membrane of the target cell. Specifically, smaller classes of TDEVs (< 200 nm) have been shown to carry diverse cargo including proteins, nucleic acids, and lipids^38,39^. Physiologically, sTDEV mediate diverse cell–cell communication^40^, alter the immune landscape^41^, and have demonstrated BBB-modulatory capacity^42–45^. Despite the suggested roles of bystander effects of tumor ablation in BBB disruption, and the established roles of sTDEV in BBB disruption and microenvironment modulation, no studies to date have investigated the role of sTDEV released by ablated tumors in pBBB disruption.

Therefore, we hypothesize that, in contrast to tumor cell death induced by standard-of-care RT, the bystander effects of H-FIRE brain tumor cell ablation are driven by small tumor-derived extracellular vesicles (sTDEV) and induce pBBB disruption following H-FIRE ablation of brain cancer cells. Herein, we compared the bystander effects of H-FIRE brain tumor ablation on BBB endothelium integrity to that of RT. We model the treatment of primary and metastatic brain cancer by treating glioma and Lewis lung carcinoma cell lines, respectively. We treat these cell lines in vitro with H-FIRE and RT, and use post-treatment supernatant exposures to model bystander effects of treatment on an in vitro BBB endothelium model. We further examined the role of sTDEV in the BBB endothelium modulation. sTDEV were isolated from post-H-FIRE and post-RT supernatants and characterized with transmission electron microscopy (TEM). We then tested their role in BBB endothelium modulation using a Transwell permeability assay, and characterized post-H-FIRE BBB-modulatory sTDEV using nanoparticle tracking analysis (NTA).

## Results

### Supernatants of H-FIRE-treated tumor cells alter cerebral endothelial cell morphology and disrupt monolayer integrity

**Fig. 1:**
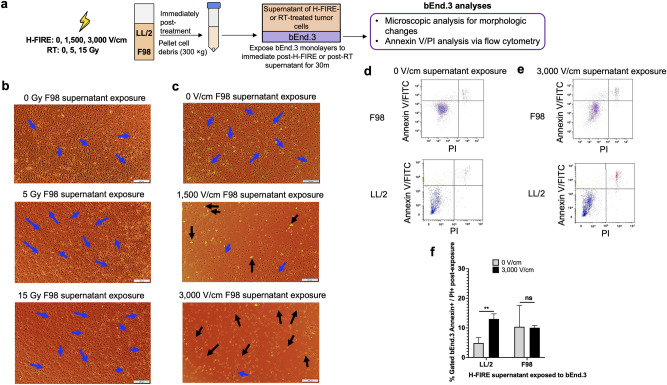
Supernatants of H-FIRE-treated tumor cells disrupt cerebral endothelial cell morphology and monolayer integrity. (**A**) F98 glioma and LL/2 Lewis lung carcinoma cells were treated with H-FIRE doses of 0, 1500, and 3000 V/cm, or with RT doses of 0, 5, and 15 Gy. Immediately following treatment, supernatants were collected and cerebral endothelial cell monolayers (bEnd.3) were exposed to post-H-FIRE or post-RT tumor cell supernatants for 30 min. Apoptosis induced by the 3000 V/cm H-FIRE-treated tumor cell supernatants relative to 0 V/cm supernatants was assessed by measuring Annexin V FITC activity and PI permeability of bEnd.3 cerebral endothelial cells after exposure to supernatants of either group. (**B**) Representative images of bEnd.3 monolayers following 30 min exposure to supernatants of RT-treated F98 glioma cells. (**C**) Representative images of bEnd.3 monolayers following 30 min exposure with supernatants of H-FIRE-treated F98 glioma cells (×10 magnification). Blue arrows identify representative morphology of adhered bEnd.3 cells, while black arrows identify cells that have lost adherence post-supernatant exposure. (**D**) Representative flow cytometry dot plots and gating of bEnd.3 cells following 30 min incubation with supernatants of 0 V/cm-H-FIRE-treated F98 or LL/2 cells. (**E**) Representative flow cytometry dot plots and gating of bEnd.3 cells following 30 min incubation with supernatants of 3,000 V/cm-H-FIRE-treated F98 or LL/2 cells. (**F**) Percentage of apoptotic (Annexin V FITC + / PI + , top right quadrant) bEnd.3 cells following 30 min exposure with supernatants of LL/2 and F98 cells treated with 0 and 3000 V/cm H-FIRE. Data are presented as mean ± SD. Statistical significance levels are noted between exposures of different supernatant treatment levels within each cell line. Statistical analysis using unpaired t tests. ns p ≥ 0.05, **p < 0.01. n ≥ 3 for all groups.

To investigate the role of bystander effects in the mechanism of pBBB disruption following H-FIRE treatment of brain tumors, we exposed supernatants from H-FIRE-treated glioma (F98) and Lewis lung carcinoma (LL/2) cell lines to monolayers of cerebral endothelial cells (bEnd.3), which were microscopically imaged for endothelial morphologic or monolayer disruption (Fig. 1A). To compare the role of ablation bystander effects in pBBB disruption between H-FIRE and RT, we also exposed bEnd.3 monolayers to supernatants of RT-treated F98 and LL/2 tumor cell lines (Fig. 1A). Supernatants of tumor cell lines treated with 0, 5, or 15 Gy had no effect on bEnd.3 monolayer integrity after 30 min of exposure (Fig. 1B, Supplementary Fig. 1). Supernatants of tumor cell lines treated with 0 V/cm H-FIRE did not visibly disrupt bEnd.3 monolayer integrity or endothelial cell morphology (Fig. 1C, Supplementary Fig. 1). Supernatants of tumor cell lines treated with 1500 V/cm H-FIRE induced partial detachment of bEnd.3 cells and moderately disrupted monolayer integrity, while supernatants of tumor cell lines treated with 3000 V/cm H-FIRE induced detachment of almost all bEnd.3 cells and completely disrupted of monolayer integrity (Fig. 1C, Supplementary Fig. 1). Representative images of bEnd.3 monolayer changes in response to exposure to supernatants of H-FIRE- and RT-treated LL/2 Lewis lung carcinoma cells are included in Supplementary Fig. 1.

To assess whether bEnd.3 cell detachment was due to cell death, we used Annexin V/FITC and propidium iodide (PI) flow cytometry^46^. We assessed apoptosis of bEnd.3 cells following a 30-min exposure to supernatants of 0 V/cm- or 3000 V/cm-H-FIRE-treated (Fig. 1D,E, respectively) F98 or LL/2 tumor cell lines. The percentage of apoptotic bEnd.3 cells, those that both bound annexin V and stained positive for PI, increased significantly when exposed to supernatants of 3000 V/cm-treated LL/2 cells (13.03%) compared to bEnd.3 cells exposure to supernatants of 0 V/cm-treated LL/2 cells (4.89%) (p < 0.01) (Fig. 1F). There was no significant difference in the percentage of apoptotic bEnd.3 cells after exposure to supernatants of 0 V/cm-treated F98 cells (10.4%) compared to after exposure to supernatants of 3000 V/cm-treated F98 cells (10.1%) (Fig. 1F)**.**

### H-FIRE ablation of brain cancer cells induces the release of vesicles that increase permeability of the BBB endothelium in vitro

The H-FIRE-specific effect of supernatants of treated tumor cells on endothelium integrity is suggestive of bystander effect-mediated mechanisms of pBBB disruption after H-FIRE-tumor ablation. We therefore hypothesized that small tumor-derived extracellular vesicles (sTDEV) are a specific bystander effector that contribute to this H-FIRE-specific bystander effect-mediated disruption of the BBB endothelium. Because supernatants of H-FIRE-treated F98 and LL/2 cells disrupted the BBB endothelium while supernatants of RT-treated tumor cells did not, we hypothesized that sTDEV isolated from supernatants of H-FIRE-treated tumor cells (sTDEV_H-FIRE_) would increase permeability of the BBB in vitro, while sTDEV isolated from supernatants of RT-treated tumor cells (sTDEV_RT_) would not.

**Fig. 2:**
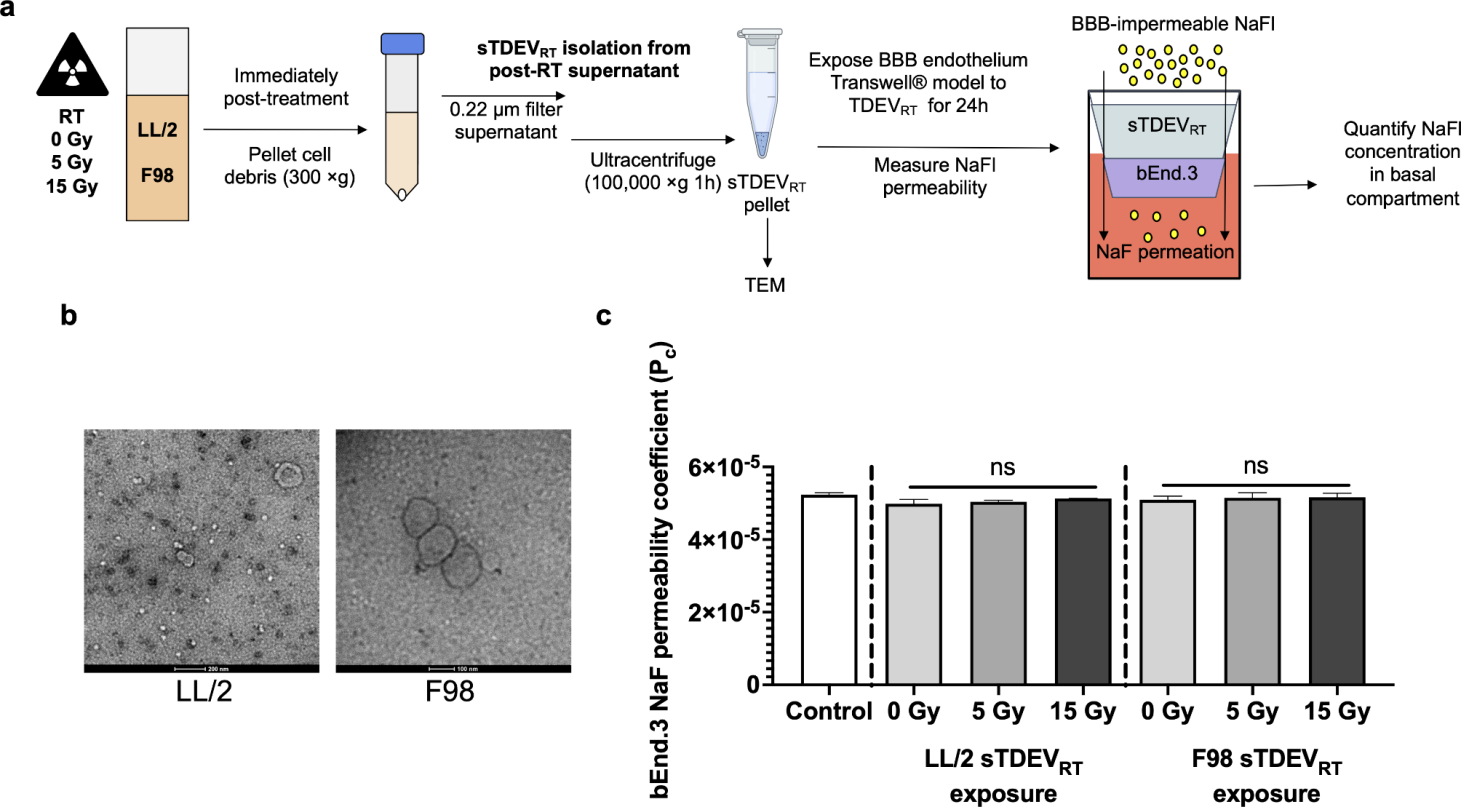
Irradiated tumor cells release sTDEV_RT_ that do not alter permeability of the BBB endothelium in vitro. (**A**) sTDEV were isolated from immediate post-RT supernatants of tumor cells irradiated with 0, 5, and 15 Gy via filtration and ultracentrifugation. TEM was used to confirm vesicular morphology and size. sTDEV_RT_ were applied to a bEnd.3 Transwell model of the BBB endothelium for 24 h, and permeability of the BBB endothelium model to NaFl was determined. Exposure of the Transwell model with complete DMEM was used as a control. (**B**) Representative TEM image of sTDEV isolated from supernatant of 15 Gy-treated LL/2 cells (sTDEV_LL/2, 15 Gy_, left) and from supernatant of 15 Gy-treated F98 cells (sTDEV_F98, 15 Gy_, right). (**C**) Permeability coefficients of bEnd.3 Transwell BBB endothelium model to NaFl after 24 h exposure to sTDEV_RT_ and complete media control. Data are presented as mean ± SD. Significant differences are noted between exposure to different sTDEV dose levels within each cell line. Statistical analysis using one-way ANOVA and Tukey’s post-hoc test. ns p ≥ 0.05, n ≥ 3 for all groups.

sTDEV_RT_ fractions were isolated from supernatants using filtration and ultracentrifugation and characterized via TEM to confirm membrane-bound vesicles with the absence of an internal nucleus (Fig. 2B). However, since the vesicles in the TEM images are not labeled with an “EV marker”, nor a western blot is provided to show the presence of traditional “EV markers” and a lack of cellular components/fragments in the isolated pellets, we proceed to call the isolated pellets from the supernatants as “sTDEV fractions” throughout the rest of the manuscript*.* sTDEV_RT_ fractions were then applied to a bEnd.3 Transwell system modelling the BBB endothelium and incubated for 24 h as negative functional controls to confirm lack of BBB endothelium-disrupting bioactivity and integrity of the Transwell model (Fig. 2A). Permeability of the BBB endothelium model to NaFl was then calculated. Exposure of the bEnd.3 Transwell model of the BBB endothelium demonstrated that none of the sTDEV_RT_ fractions (sTDEV_0Gy_, sTDEV_5Gy_, sTDEV_15Gy_) significantly altered permeability of our BBB endothelium model after 24 h of exposure (Fig. 2C).

**Fig. 3:**
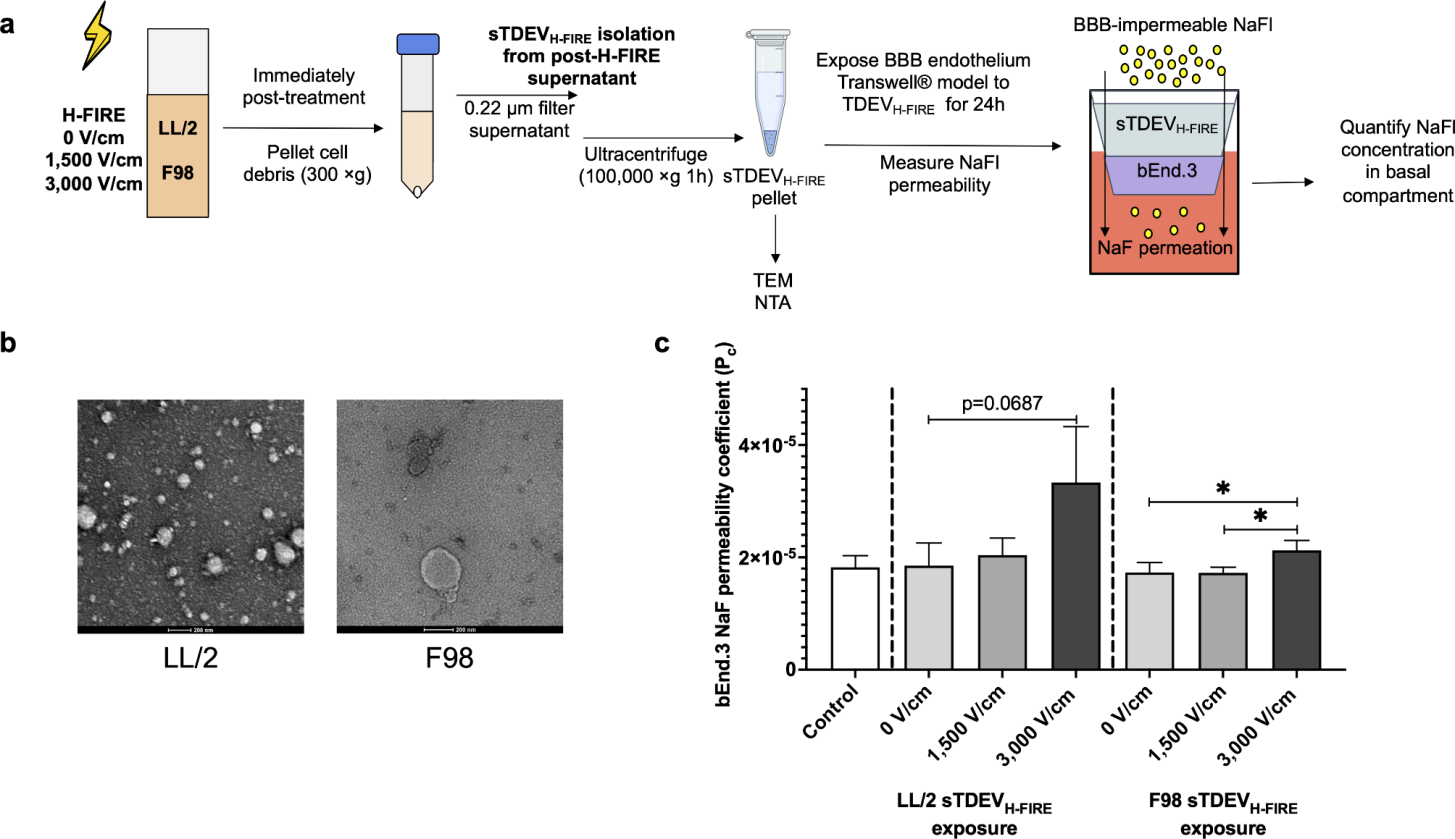
sTDEV released by 3,000 V/cm H-FIRE-treated tumor cells increase permeability of the BBB endothelium in vitro. (**A**) sTDEV were isolated from immediate post-H-FIRE supernatants of tumor cells treated with 0, 1500, and 3000 V/cm H-FIRE via filtration and ultracentrifugation. TEM was used to confirm vesicular morphology and size. sTDEV_H-FIRE_ were applied to a bEnd.3 Transwell model of the BBB endothelium for 24 h, and permeability of the BBB endothelium model to NaFl was determined. Exposure of the Transwell model with complete DMEM was used as a control. (**B**) Representative TEM image of sTDEV isolated from supernatant of 3000 V/cm-treated LL/2 cells (sTDEV_LL/2, 3,000 V/cm_, left) and from supernatant of 3000 V/cm-treated F98 cells (sTDEV_F98, 3,000 V/cm_, right). (**C**) Permeability coefficients of bEnd.3 Transwell BBB endothelium model to NaFl after 24 h exposure with sTDEV_H-FIRE_ and complete media control. Data are presented as mean ± SD. Significant differences are noted between exposure with different sTDEV dose levels within each cell line. Statistical analysis using one-way ANOVA with Tukey’s post-hoc test. ns p ≥ 0.05, *p < 0.05, n ≥ 3 for all groups.

To determine whether the observed bystander effect-mediated disruption of bEnd.3 monolayers following exposure to supernatants of H-FIRE-treated cancer cells is mediated by sTDEV fractions, we exposed the bEnd.3 Transwell model of the BBB endothelium to sTDEV_H-FIRE_ fractions (Fig. 3A). TEM of sTDEV fractions isolated from supernatants of 3000 V/cm-treated LL/2 and F98 cells (TDEV_3000 V/cm_) confirm that sTDEV_H-FIRE_ fractions are membrane-bound and lack an internal nucleus (Fig. 3B). Exposure of the bEnd.3 Transwell model to sTDEV_LL/2, 3000 V/cm_ fraction did not affect the permeability of the BBB endothelium model to NaFl after 24 h of exposure compared to sTDEV_LL/2, 0 V/cm_ (p = 0.069) (Fig. 3C). sTDEV_F98, 3000 V/cm_ fractions significantly increased permeability of the BBB endothelium model to NaFl after 24 h of exposure compared to sTDEV_F98, 0 V/cm_ and sTDEV_F98, 1500 V/cm_ (Fig. 3C).

### H-FIRE ablation of glioma cells decreases the release of sTDEV

**Fig. 4:**
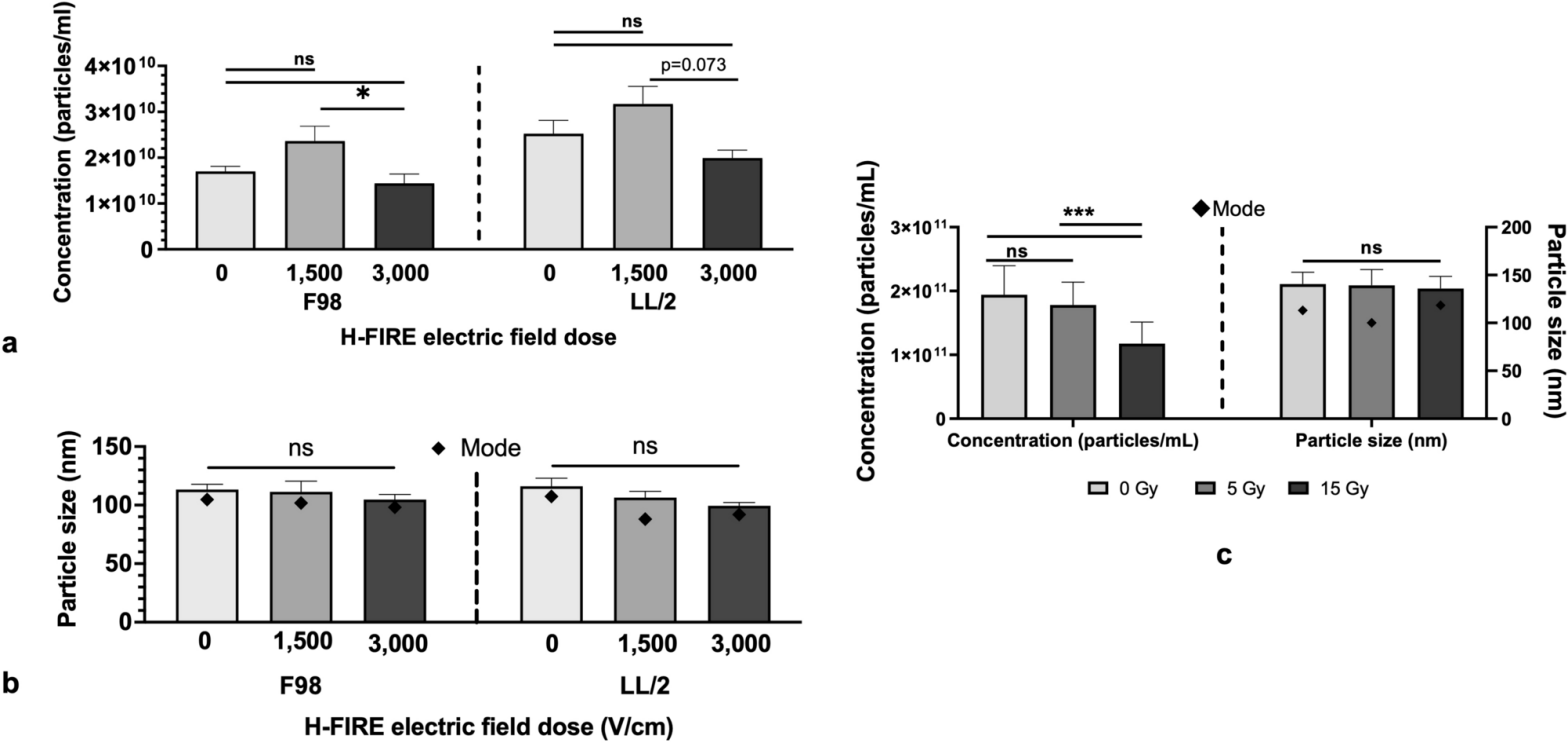
H-FIRE reduces the release of sTDEV that increase permeability of the BBB endothelium in vitro. sTDEV were isolated from immediate post-H-FIRE supernatants of F98 glioma and LL/2 Lewis lung carcinoma cells treated with 0, 1500, and 3000 V/cm H-FIRE, and F98 glioma cells treated with 0, 5, and 15 Gy RT, via filtration and ultracentrifugation. Nanoparticle Tracking Analysis (NTA) was used to quantify concentrations of isolated sTDEV and mean and mode particle sizes. (**A**) NTA of sTDEV_H-FIRE_ concentrations. Data are presented as mean ± SE. Significant differences are noted between different H-FIRE dose levels within each cell line. Statistical analysis using one-way ANOVA with Tukey’s post-hoc test. ns p ≥ 0.05, *p < 0.05, n ≥ 3 for all groups. (**B**) NTA of sTDEV_H-FIRE_ mean and mode particle sizes. Data are presented as mean ± SE. Significant differences between mean particle sizes are noted between different H-FIRE dose levels within each cell line. Statistical analysis using one-way ANOVA with Tukey’s post-hoc test. ns p ≥ 0.05, n ≥ 3 for all groups. (**C**) NTA of sTDEV released by F98 glioma cells treated with RT (sTDEV_F98, RT_) showing particle concentrations in the sTDEV_RT_ fractions, and mean and mode particle sizes. Data are presented as mean ± SE. Significant differences are noted between different RT dose levels. Statistical analysis using one-way ANOVA with Tukey’s post-hoc test. ns p ≥ 0.05, *p < 0.05, n ≥ 3 for all groups.

To determine how H-FIRE impacts the number of sTDEVs in the fractions released into the supernatants, we measured the particle concentration in the sTDEV_H-FIRE_ fractions via nanoparticle tracking analysis (NTA). NTA demonstrates that, for both cancer cell lines, 1500 V/cm H-FIRE treatment insignificantly increased the release of sTDEV compared to sham treatment (Fig. 4A). Treatment with 3000 V/cm H-FIRE insignificantly decreased the release of sTDEV_LL/2, 3000 V/cm_ compared to sTDEV_LL/2, 1500 V/cm_ (Fig. 4A). Treatment with 3000 V/cm H-FIRE significantly decreased the release of sTDEV_F98, 3000 V/cm_ relative to sTDEV_F98, 1500 V/cm_, but not relative to sTDEV_F98, 0 V/cm_ (Fig. 4A). Mean and mode particle sizes of sTDEV_H-FIRE_ were not significantly different between any H-FIRE doses (Fig. 4B). Compared to concentrations of sTDEV_F98_ after 0 Gy RT (1.14 × 10^11^ particles/mL), sTDEV concentrations after 15 Gy (1.14 × 10^10^ particles/mL) were significantly decreased (p < 0.001) (Fig. 4C). Mean particle size of sTDEV released after 0, 5, and 15 Gy RT did not significantly change between different RT doses (Fig. 4C). Mode particle size of sTDEV released after 0, 5, and 15 Gy RT was significantly different between 5 and 15 Gy (p < 0.01) (Fig. 4C).

## Discussion

Following H-FIRE ablation of brain tumors, the pBBB is focally and transiently disrupted^18,20,21^. This enhanced pBBB permeability following H-FIRE ablation of brain tumors may be exploited for improved delivery of systemic therapeutics to treat infiltrative tumor cells beyond the borders of tumor ablation. This pBBB disruption after treatment of brain tumors is not unique to H-FIRE: a standard-of-care brain tumor treatment modality, RT, also induces disruption of the pBBB, the mechanisms of which has been attributed to direct exposure of pBBB cells to radiation and the subsequent induction of apoptosis, ultrastructural changes, and senescence in BBB endothelial cells^22,23^. However, the mechanisms of the pBBB disruption following H-FIRE brain tumor ablation are not well understood. Our results indicate that supernatants of H-FIRE-treated brain cancer cells disrupt the BBB endothelium in an H-FIRE-specific and dose-dependent manner under these experimental conditions. This involves the disruption of endothelial monolayer integrity via detachment of endothelial cells, the extent of which is dependent on the applied electric field of the supernatants. Supernatants of irradiated tumor cells do not disrupt cerebral endothelial cell monolayer integrity in the same way as H-FIRE supernatants. We further demonstrate that the observed endothelial monolayer disruption after exposure to supernatants of H-FIRE-treated tumor cells is not significantly attributable to endothelial cell apoptosis. Although supernatants of 3000 V/cm H-FIRE-treated LL/2 cells induced significant increases in bEnd.3 cell apoptosis, the level of disruption of bEnd.3 monolayer integrity observed via microscopy is not sufficiently explained by the relatively small increase in apoptotic bEnd.3 cells post-exposure. These data suggest that supernatants of H-FIRE-treated LL/2 cells may contain factors that induce apoptosis in recipient cells, but additional mechanisms seem to be at play in the physical detachment of the bEnd.3 cells post-exposure. The mechanisms underlying the slight increase in endothelial cell apoptosis post-supernatant exposure will be the subject of future investigations. In characterizing the supernatants of H-FIRE- and RT-treated cancer cells, transmission electron microscopy (TEM) demonstrates that H-FIRE- and RT-treated tumor cell lines release small tumor-derived extracellular vesicles (sTDEV_H-FIRE_ and sTDEV_RT_, respectively). Using a Transwell model of the BBB endothelium, we demonstrate that sTDEV fractions released by 3000 V/cm H-FIRE-treated glioma cells, but not irradiated cells, increase cerebral endothelium permeability. This supports our hypothesis that bystander effects of H-FIRE brain tumor cell ablation, specifically the release of sTDEV, uniquely disrupt the integrity of the pBBB endothelium following H-FIRE brain tumor cell ablation. Further analysis of sTDEV_H-FIRE_ fractions demonstrates decreased release of sTDEV after the 3000 V/cm H-FIRE treatment, suggesting that the observed effect is likely due to H-FIRE-induced alterations in sTDEV cargo in the isolated sTDEV fractions rather than changes in TDEV concentration.

Taken together, our data suggest that sTDEV fractions disrupt the BBB endothelium after H-FIRE ablation of brain tumors in novel bystander effect-mediated mechanism of pBBB disruption that is unique to H-FIRE compared to standard-of-care RT. The fact that there are fewer sTDEV_3000 V/cm_ compared to sTDEV_1500 V/cm_ and sTDEV_0V/cm_, but sTDEV_3000 V/cm_ fractions increase permeability of the BBB endothelium in vitro, suggests that H-FIRE alters cargo of the sTDEV at the 3000 V/cm dose. Based on the NTA, the size of the sTDEV does not change with dose (Fig. 4B), suggesting that the subtype of TDEV does not change with H-FIRE electric field dose. The mean and mode particle sizes of sTDEV_H-FIRE_ within the 100–150 nm range suggest that sTDEV_H-FIRE_ are comprised primarily of exosomes, a specific subtype of TDEV^47^. However, size alone is insufficient to conclude vesicular subclasses, and therefore future studies should seek to characterize subpopulations of sTDEV with markers for exosomes and microvesicles. Overall, because TDEV_3000 V/cm_ fractions increased BBB endothelium permeability in vitro with fewer sTDEV than sTDEV_1500 V/cm_ and sTDEV_H-FIRE, sham_, our data suggest that H-FIRE alters the cargo of sTDEV in a way that disrupts the function of the BBB endothelium. Future work is warranted to characterize the timescale of proteomic, nucleic acid, and lipidomic payloads of sTDEV_H-FIRE_ to identify the mechanisms of sTDEV_H-FIRE_-mediated BBB endothelium disruption.

Although our data demonstrate the effects of sTDEV_H-FIRE_ fractions on the BBB endothelium in vitro, the lack of characterization of sTDEV_H-FIRE_ effects on co-culture models of the BBB, including other critical cellular BBB components such as astrocytes, is a limitation of this study. Additionally, future investigations into the precise mechanisms underlying the disruption of cerebral endothelial cells, such as the potential disruption of adhesion proteins or tight junction complexes, is warranted. Interestingly, the fact that supernatants of 1500 V/cm-treated tumor cells visibly disrupted cerebral endothelial monolayer integrity, but sTDEV_1500_ isolated from those supernatants did not increase permeability of the Transwell model, suggests that other non-sTDEV bystander effectors may contribute to the mechanism of cerebral endothelium disruption at lower H-FIRE doses. It also must be considered that although we have previously demonstrated the respective sub-ablative and ablative effects of the 1500 V/cm and 3000 V/cm H-FIRE doses using this cancer cell suspension model, the altered morphology of cells in suspension increases the specific electric field thresholds required to induce these sub-ablative and ablative effects relative to cellular morphology in vivo^48,49^. Therefore, future studies should seek to determine the specific electric field thresholds required to induce the described sTDEV functional alterations in tumor cells with physiologic morphology. Our study specifically explores the role of sTDEV_H=FIRE_ in disruption of the peritumoral BBB following ablation of the tumor bulk. Given the extensive literature implicating sTDEV in modulation of the tumor microenvironment, it is likely that these sTDEV_H-FIRE_ fractions play additional roles in altering the tumor microenvironment following tumor ablation, with subsequent implications for post-treatment tumor response. Future studies will seek to characterize these functions with respect to anti-tumor or pro-tumor effects of sTDEV_H-FIRE_ in the tumor microenvironment following H-FIRE treatment. Further, it is known that there are differences in the cellular architecture of healthy BBB and tumor-associated BBB, or the blood-tumor barrier (BTB), which may be relevant to the tumor-brain parenchyma interface where pBBB disruption is observed after H-FIRE ablation of brain tumors^50,51^. Therefore, future work will evaluate the effects of sTDEV_H-FIRE_ on in vitro and in vivo models of the BBB and BTB. Further experiments characterizing the timescale of sTDEV_H-FIRE_-mediated BBB disruption, molecular subpopulations and payloads of sTDEV_H-FIRE_, and evaluating the effects of sTDEV_H-FIRE_ on the in vivo BBB, are ongoing.

Our work is the first to implicate tumor ablation-induced alterations of sTDEV in the disruption of the pBBB. Indeed, recent studies of H-FIRE tumor treatment effects have demonstrated the release of extracellular factors and signaling molecules from ablated tumor cells such as reactive oxygen species, ATP, and various damage-associated molecular patterns (DAMPs)^31,32^. Many such extracellular factors are known to influence BBB permeability and physiology in non-tumor-bearing brain environments^33–36^. Specifically, Sheybani et al*.* demonstrated that an alternative tumor ablation therapeutic, focused ultrasound hyperthermia, augmented the release of sTDEV from treated glioma cells^37^. Small extracellular vesicles have been otherwise shown to modulate the BBB in multiple contexts in non-tumor-bearing brains^42–44^. Likewise, sTDEV released by glioma cells have been shown to modulate BBB functionality. Zhao et al*.* demonstrated that sTDEV released by hypoxic glioblastoma cells increase permeability of the BBB compared to sTDEV released by normoxic glioblastoma cells^45^. Our results are consistent with these findings that other energy-based tumor ablation modalities have been shown to alter the release of sTDEV, and that sTDEV have been otherwise implicated in BBB modulation. Our work is the first to draw a direct link between the H-FIRE tumor ablation sequela and the disruption of the pBBB, demonstrating that a specific bystander effector of H-FIRE treatment, sTDEV, directly facilitates the H-FIRE-specific bystander effect-mediated disruption of the BBB endothelium in vitro. These data suggest that this mechanism is unique to H-FIRE when compared to standard-of-care RT, which is consistent with the fact that mechanisms of RT-induced pBBB disruption depend on direct radiation exposure of pBBB cells^22,23^.

A comprehensive understanding of the mechanisms underlying the transient pBBB disruption that occurs secondary to H-FIRE ablation of brain tumors is critical for the rational design of multimodal therapeutic approaches. Such approaches may employ H-FIRE ablation of the bulk tumor, followed by mechanism-driven exploitation of the post-ablation window of pBBB disruption for the improved delivery of systemic therapeutics. This will enable the treatment of infiltrative neoplastic margins which, if left untreated, otherwise lead to tumor recurrence. Taken together, these data suggest that bystander effects of H-FIRE tumor ablation disrupt the BBB endothelium. Specifically, H-FIRE ablation of brain cancer cells appears to alter sTDEV fractions to modulate BBB endothelium integrity in vitro, suggesting an H-FIRE-specific mechanism of sTDEV-mediated BBB endothelium disruption after treatment of the tumor. This data provides improved understanding of potential mechanisms of post-H-FIRE ablation BBB endothelium disruption, which may pave the way for the rational design of post-ablation drug delivery approaches to not only treat the bulk tumor volume via ablation with H-FIRE, but to mechanistically exploit the window of pBBB disruption to treat infiltrative tumor cell populations following ablation.

## Methods

### Cell culture

Murine Lewis lung carcinoma cell line LL/2 (LLC1) (ATCC, CRL-1642), rat glioma cell line F98 (ATCC, CRL-2397) and murine cerebral endothelial cell line bEnd.3 (ATCC, CRL-2299) were grown in DMEM (ATCC, 30-2002) supplemented with 10% standard fetal bovine serum (Thermo Fisher Scientific, Gibco) and 1% penicillin–streptomycin (Thermo Fisher Scientific, Gibco). Cells were incubated at 37 °C in a humidified environment containing 5% CO_2_ and subcultured regularly. All cells used were between passages 5 and 14, and when necessary, were detached for use in experiments using TrypLE Express Enzyme at room temperature (Thermo Fisher Scientific, Gibco).

### H-FIRE treatment of tumor cell lines and supernatant exposure of cerebral endothelial cell monolayers

bEnd.3 cells were grown to 90% confluence in 12-well plates (USA Scientific) in DMEM supplemented with 10% standard fetal bovine serum (Thermo Fisher Scientific, Gibco) and 1% penicillin–streptomycin (Thermo Fisher Scientific, Gibco). Cells were incubated at 37 °C in a humidified environment containing 5% CO_2_. Media was refreshed every two days. LL/2 and F98 cells were grown in to 90% confluence in DMEM supplemented with 10% standard fetal bovine serum (Thermo Fisher Scientific, Gibco) and 1% penicillin–streptomycin (Thermo Fisher Scientific, Gibco). At near confluence and following detachment with TrypLE, LL/2 and F98 cells were washed and resuspended in a 5.5:1 ratio of low-conductivity sucrose solution (85 g sucrose, 3 g glucose, 7.25 ml DMEM, and 992.75 ml DI water) to unsupplemented DMEM to a concentration of 1.25 × 10^6^ cells/ml. 800 μl of cell suspension were aliquoted into 4 mm gap sterile electroporation cuvettes (USA Scientific). H-FIRE pulse parameters were delivered to cuvettes using a custom-built H-FIRE pulse generator (VoltMed Inc, Blacksburg, VA). H-FIRE treatments consisted of 200 bursts of bipolar pulses delivered at a frequency of 1 burst per second. One burst of bipolar pulses consisted of a 2 μs positive pulse, a 5 μs interphase delay, a 2 μs negative pulse, and a 5 μs interpulse delay (2-5-2 pattern) which was repeated until a total energized time of 100 μs was achieved. Treatment voltage and current were monitored using an oscilloscope. H-FIRE treatments consisting of these parameters were delivered at electric field magnitudes of 0, 1500, and 3000 V/cm. We selected these doses based on our previous work demonstrating sub-ablative (1500 V/cm), and ablative (3000 V/cm) effects on these cell lines^48^. Following treatment, cell suspensions were kept at 37 °C for 20 min prior to centrifugation and supernatant collection. Suspensions were then centrifuged at 300 × g for 6 min, and 800 μl of supernatant were collected from each H-FIRE suspension. Media was removed from bEnd.3 cells and monolayers were rinsed with Hanks Balanced Salt Solution (Thermo Fisher Scientific, Gibco). BEnd.3 monolayers were exposed to 800 μl of supernatant from each H-FIRE-treated cancer cell suspension in triplicates, and plates were incubated at 37 °C in a humidified environment containing 5% CO_2_ for 30 min. Following exposure, bEnd.3 monolayers were imaged and photographed using an inverted microscope (Olympus IX37) under bright field 10X magnification. All H-FIRE treatments were performed in triplicates.

### Irradiation of tumor cell lines and supernatant exposure of cerebral endothelial cell monolayers

bEnd.3 cells were grown to 90% confluence in 12-well plates (USA Scientific) in DMEM supplemented with 10% fetal bovine serum (Thermo Fisher Scientific, Gibco) and 1% penicillin–streptomycin (Thermo Fisher Scientific, Gibco). Cells were incubated at 37 °C in a humidified environment containing 5% CO_2_. Media was refreshed every two days. At near confluence and following detachment with TrypLE, LL/2 and F98 cells were washed and resuspended in DMEM supplemented with 10% fetal bovine serum (Thermo Fisher Scientific, Gibco) and 1% penicillin–streptomycin (Thermo Fisher Scientific, Gibco) to a concentration of 1.25 × 10^6^ cells/ml. 800 μl of cell suspension were aliquoted into 6-well plates for RT treatment. Three wells of each cell line were treated using a 6 MV X-ray medical linear accelerator with a single fraction dose of either 0, 5, or 15 Gy (Edge, Varian Medical Systems, Inc.). The setup of the plates on the treatment couch and the calculation of the appropriate number of monitor units (MUs) to deliver the desired radiation dose to the cells were performed as previously described^52^. Briefly, the accelerator gantry and collimator were set to 0°. The field size was set to 20 × 20 cm^2^. The MUs were calculated using source to axis distance geometry. Each plate was placed between a 5 cm (bottom) and a 1 cm (top) water equivalent material on the treatment couch. This placed the cells at an average depth of 1.5 cm (treatment depth). The prescribed doses were delivered at a 600 MU/min rate. Following irradiation, cancer cells were incubated for 20 min at 37 °C prior to centrifugation and supernatant collection. Media was then removed from the wells and centrifuged at 300 × g for 6 min. 800 μl of supernatant were collected from each radiation suspension. Media was removed from bEnd.3 cells and monolayers were rinsed with Hanks Balanced Salt Solution (Thermo Fisher Scientific, Gibco). Bend.3 monolayers were exposed to 800 μl of supernatant from each irradiated cancer cell suspension in triplicates, and plates were incubated at 37 °C in a humidified environment containing 5% CO_2_ for 30 min. Following exposure, bEnd.3 monolayers were imaged and photographed using an inverted microscope (Olympus IX37) under bright field 10X magnification.

### Flow cytometry apoptosis assay of cerebral endothelial cells exposed to supernatants of H-FIRE-treated tumor cell lines

Following a 30-min exposure of bEnd.3 monolayers to supernatants of H-FIRE-treated tumor cell lines and microscopic imaging, supernatants were aspirated from bEnd.3 wells to remove detached bEnd.3 cells after exposure to 0 V/cm and 3000 V/cm H-FIRE-treated tumor cell supernatants. Any bEnd.3 cells remaining in the well were detached with Trypsin–EDTA (0.25%) (Thermo Fisher Scientific, Gibco), rinsed with complete DMEM, and combined with the corresponding aspirate. Aspirates were centrifuged at 300 × g for 6 min, and bEnd.3 cell pellets were resuspended in complete DMEM and placed in a bead bath for 20 min. Cell suspensions were centrifuged at 300 × g for 6 min, washed with PBS, and centrifuged at 300 × g for 6 min. bEnd.3 cells were analyzed for apoptosis using the eBioscience™ Annexin V Apoptosis Detection Kit FITC and Propidium Iodide (Thermo Fisher Scientific, Invitrogen) per manufacturer’s protocol. Triplicate bEnd.3 wells were collected per supernatant exposure condition, and cells from each bEnd.3 well were analyzed by flow cytometry in duplicates (Cytoflex, Beckman Coulter).

### sTDEV isolation

Twenty-four hours prior to H-FIRE or RT treatment of tumor cell lines, media was removed from LL/2 and F98 cultures. Monolayers were rinsed 5 times with Hank’s Balanced Salt Solution (Thermo Fisher Scientific, Gibco), and media was replaced with DMEM supplemented with 10% exosome-depleted fetal bovine serum (Thermo Fisher Scientific, Gibco) and 1% penicillin–streptomycin (Thermo Fisher Scientific, Gibco). LL/2 and F98 cancer cell lines were then treated with H-FIRE or RT protocols as described above. In each H-FIRE treatment, 1.0 × 10^6^ cells were treated at a concentration of 1.25 × 10^6^ cells/ml. Following H-FIRE or RT treatment of cancer cell suspensions, the entire cell suspension was collected and centrifuged at 300 × g for 6 min to remove cell debris. The entire supernatant was then syringe filtered through pre-wetted 0.22 μm polyester syringe filters. Filtration eluates were ultracentrifuged at 100,000 × g for 1 h at 4 ºC (Beckman Optima TLX Ultracentrifuge, TLA 120.2 rotor). Supernatants were removed and 200 μL of either HEPES buffer (100 mM NaCl, 20 mM HEPES sodium salt, 4 mM KCl in milliQ water) or pre-warmed complete DMEM was added to sTDEV pellets and left overnight at 4ºC. Pellets were triturated to resuspend the sTDEV. HEPES sTDEV suspensions were used for transmission electron microscopy (TEM) and nanoparticle tracking analysis (NTA). TDEVs suspended in complete DMEM were used for BBB permeability experiments.

### Transmission electron microscopy (TEM)

Formvar-coated 200 mesh copper grids (Electron Microscopy Sciences, Hatfield PA) were glow discharged on a Pelco glow discharge unit (Pelco, Fresno, CA) at 0.29 mBAR for 1 min. Grids were coated with 0.1% poly-l-lysine for 1 min. Excess solution was removed with Whatman #1 filter paper (Whatman PLC, Maidstone UK). Grids were washed twice with 10 μL milli-Q (Millipore Sigma) water and blotted with filter paper to remove. Grids were dried overnight at room temperature, and 10 μL of sTDEV suspension were added to the grid for 5 min. Excess solution was removed with filter paper, and the sample was negatively stained with 10 μL Uranyless stain (Electron Microscopy Sciences) for 1 min. Excess solution was wicked away with filter paper. Grids were dried at room temperature overnight before TEM. Imaging was performed on a FEI Tecnai G20 Biotwin TEM (FEI Company, Hillsboro, OR) at 120 kV using an Eagle 4 K HS camera (GATAN, Pleasanton, CA).

### In vitro BBB permeability assay

BEnd.3 cells were seeded on the apical side of 12 mm, 3.0 μm pore, polyester Transwell inserts (Corning) at a density of 4 × 10^4^ cells/cm^2^ and grown for 5 days to confluence. Apical and basal chamber media was changed every two days, and monolayers were grown until confluence on day 5^53–56^. On day 5, Transwell inserts containing bEnd.3 cells were placed in new 12-well plates (USA Scientific). Media was removed from the apical and basal sides of bEnd.3-seeded Transwells. sTDEVs, isolated from the treatment of 1.0 × 10^6^ cells according to the methods described above, were suspended in complete DMEM were added to the apical compartment, and 1 mL of complete DMEM was added to the basal compartment. Each bEnd.3 Transwell was exposed to the entire sTDEV isolate from one H-FIRE or one RT treatment, meaning each well was exposed to the total sTDEVs isolated after the treatment of 1.0 × 10^6^ tumor cells. We normalized these exposures to the number of treated cells to model the treatment of a tumor of a consistent size with different treatment doses. Exposure of bEnd.3 cells with 200 μL complete DMEM was used as an additional control. bEnd.3 cells were incubated with sTDEV or control media at 37 °C in a humidified environment containing 5% CO_2_ for 24 h. All exposures were performed in triplicates. After 24 h of exposure, sTDEV suspensions were removed from the apical chamber and media was removed from the basal chamber. 500 μL of 10 μg/mL sodium fluorescein salt (NaFl) in complete DMEM were added to the apical chamber, and 1 mL complete DMEM was added to the basal chamber and incubated for 1 h at 37 °C in a humidified environment containing 5% CO_2_. Following 1 h of dye incubation, the fluorescence of the basal chamber was measured using a microplate reader (485 nm excitation, 528 nm emission). Permeability coefficients (P_c_) were calculated using Eq. (1).

1 $${\text{P}}_{{\text{c}}} = \left( {{\text{V}}_{{\text{r}}} {\text{x C}}_{{\text{f}}} } \right)/\left( {{\text{C}}_{{\text{i}}} {\text{x A x t}}} \right)$$

V_r_ is the basal chamber volume (mL), C_f_ is the final apical dye concentration (μM), C_i_ is the initial apical dye concentration (μM), A is the membrane growth area (cm^2^), and t is the assay time (seconds).

### Nanoparticle tracking analysis (NTA)

NTA of isolated sTDEV was performed on a Nanosight NS300 (Malvern Panalytical, Malvern, UK) at room temperature. sTDEV suspended in HEPES buffer were diluted 1:100 in degassed HEPES buffer (100 mM NaCl, 20 mM HEPES sodium salt, 4 mM KCl in milliQ water, degassed overnight at room temperature), and were sonicated for 30 s at room temperature. Suspensions were loaded into a syringe pump (Malvern Panalytical) and loaded into the Nanosight large volume flow cell at a constant flow rate (0.02 ml/min). Each sample was analyzed using a 488 nm laser with 5 consecutive 1 min video recordings, with a 30 s delay between video captures. Videos were analyzed in the NTA software (Version 3.4).

### Statistical analysis

All experiments were performed with at least three replicates per experiment. Results are presented as mean ± standard deviation (SD) unless otherwise noted. Statistical analysis was performed on Graphpad Prism 9 for Mac OS, (GraphPad Software, San Diego, California USA, www.graphpad.com).

## Supplementary Information

Supplementary Information.

## Data Availability

The original data presented in the study are available from the corresponding author upon reasonable request. Additional requests for supporting information may be directed to the corresponding author.
