# Supplementary material for: Tumor-derived extracellular vesicles disrupt the blood–brain barrier endothelium following high-frequency irreversible electroporation
Source: Sci Rep. 2024 Nov 18;14:28533. doi: 10.1038/s41598-024-79019-5 (PMC11574144; doi:10.1038/s41598-024-79019-5)
Supplement: Supplementary file 1 — Supplementary Information. [file 41598_2024_79019_MOESM1_ESM.pdf]

Tumor-derived extracellular vesicles disrupt the blood-brain barrier endothelium following high-frequency irreversible electroporation

Kelsey R. Murphy*, Kenneth N. Aycock, Spencer Marsh, Alayna N. Hay, Ilektra Athanasiadi, Shay Bracha, Christine Chang, Robert Gourdie, Rafael V. Davalos, John H. Rossmeisl, Nikolaos G. Dervisis

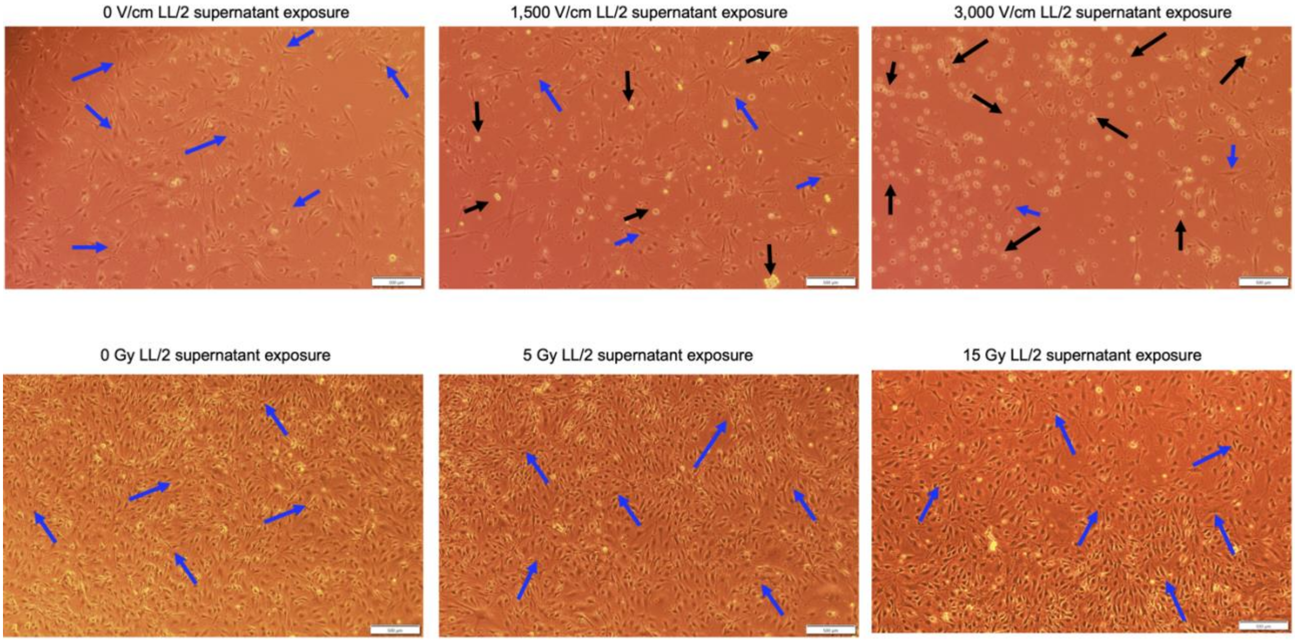

Supplemental Figure 1. Representative images of disruption of cerebral endothelial cell morphology and monolayer integrity induced by supernatants of H-FIRE- and RT-treated LL/2 Lewis lung carcinoma cells. LL/2 Lewis lung carcinoma cells were treated with H-FIRE doses of 0, 1,500, and 3,000 V/cm and RT doses of 0, 5, and 15 Gy. Immediately following treatment, supernatants were collected and cerebral endothelial cell monolayers (bEnd.3) were exposed to post-H-FIRE or post-RT tumor cell supernatants for 30 minutes. The top row depicts representative images of bEnd.3 monolayers following 30 minute exposure with supernatants of H-FIRE-treated LL/2 Lewis lung carcinoma cells (10X magnification). The bottom row depicts representative images of bEnd.3 monolayers following 30 minute exposure with supernatants of RT-treated LL/2 Lewis lung carcinoma cells (10X magnification). Blue arrows identify representative morphology of adhered bEnd.3 cells, while black arrows identify cells that have lost adherence post-supernatant exposure.

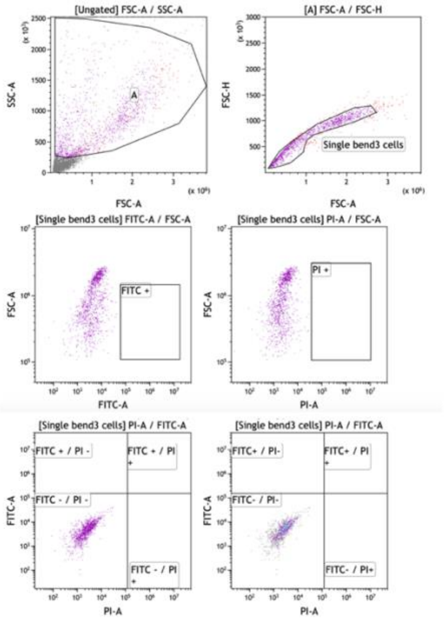

Supplemental Figure 2. Flow cytometry gating strategy for Annexin V/PI assay of bEnd.3 cells exposed to supernatants of 0 V/cm or 3,000 V/cm H-FIRE-treated F98 or LL/2 cells.
